# Telemonitoring during Exercise Training in Cardiac Telerehabilitation: A Review

**DOI:** 10.31083/j.rcm2404104

**Published:** 2023-04-04

**Authors:** Mai Shimbo, Eisuke Amiya, Issei Komuro

**Affiliations:** ^1^Department of Cardiovascular Medicine, The University of Tokyo Hospital, 113-8655 Tokyo, Japan; ^2^Department of Computational Diagnostic Radiology and Preventive Medicine, The University of Tokyo Hospital, 113-8655 Tokyo, Japan; ^3^Department of Therapeutic Strategy for Heart Failure, The University of Tokyo, 113-8655 Tokyo, Japan

**Keywords:** cardiac telerehabilitation, real-time telemonitoring, real-time supervision, synchronous, cardiovascular diseases, exercise

## Abstract

Comprehensive cardiac rehabilitation (CR) is promising strategy for various cardiovascular diseases. Despite these benefits and the recommendation, adherence to outpatient CR remains low. Home-based CR with telemedicine (tele-CR) is emerging concept that is a good alternative to conventional center-based CR. With the development of e-health and e-Cardiology, real-time telemonitoring of patients’ parameters such as vital signs and supervising by healthcare professionals during exercise training via internet might make it possible for safe and effective tele-CR to be performed. Therefore, the present study reviews the literature to summarize the current situation and methodology of patient telemonitoring in tele-CR.

## 1. Introduction: Why is Cardiac Telerehabilitation Needed?

Comprehensive cardiac rehabilitation (CR) is an integrated, critical, and evidence-based intervention for the secondary prevention of various cardiovascular diseases. It is strongly recommended by the European Society of Cardiology, American Heart Association, and American College of Cardiology [1, 2]. CR reduces the mortality rate and increases exercise tolerance and quality of life in patients with cardiovascular disease [3, 4]. Despite these benefits and recommendations, adherence to outpatient CR remains low [5, 6]. The barriers to CR participation include problems with resources (e.g., distance or costs) as well as logistical and psychological matters (e.g., lack of transportation, family support, motivation, or encouragement) [7]. Furthermore, the coronavirus disease 2019 (COVID-19) pandemic had a great impact on the care of patients with cardiovascular disease as well as on CR [8]. Center-based outpatient phase 2 CR (CBCR) has been interrupted in many areas owing to recommendations for physical distancing [9]. Thus, an alternative system to CBCR is required.

Home-based CR (HBCR) is defined as a structured program that includes exercise training or patients’ education with monitoring, follow-up visits, or telephone calls from staff or self-monitoring diaries which are carried out in various settings out of hospitals, including home [10]. Although it is often difficult to compare the effectiveness between HBCR and CBCR due to many affecting factors, little difference was seen in total mortality up to 12 months, exercise capacity or health-related quality of life up to 24 months in patients with cardiovascular disease [11]. Recently, the development of e-health and digital devices has increased the feasibility of telemedicine. HBCR with telemedicine (tele-CR) has been reported to be a good alternative system to CBCR [12, 13]. Unlike CBCR, the lack of close vital monitoring or supervision by neighboring healthcare professionals in tele-CR may pose risks in CR sessions, thus raising safety concerns. To solve this problem, tele-CR with telemonitoring of vital signs and participants’ conditions should be adopted as the novel concept. With the development of worldwide networks by internet, real-time monitoring during tele-CR has become possible [14].

## 2. Unmet Needs for Monitoring and Supervision in tele-CR

The HF-ACTION (Heart Failure: A Controlled Trial Investigating Outcomes of Exercise Training) trial demonstrated that exercise training that includes home-based self-exercise after a 3-month CBCR barely reduced the rates of all-cause mortality or hospitalization and cardiovascular mortality or heart failure hospitalization only after adjusting for highly prognostic factors [15]. The low level of participation and exercise volume in home-based exercise training was considered to be related to this unfavorable result of HF-Action. Subanalysis revealed that moderate levels of exercise volume (3–7 metabolic equivalent of task (MET)-h (the product of exercise intensity and the hours of exercise) per week) would result in better clinical outcomes [16]. Moreover, there are many outpatient programs, including exercise training, and educational programs recommended to manage cardiovascular diseases; however, the use of systems that rely on patients themselves can lead to inadequate performance and makes it difficult to ensure quality. Thus, close monitoring, follow-up, and support by healthcare professionals are crucial to maintaining high compliance in self-care and exercise training. Telemonitoring may provide access to healthcare services and communication to maintain a good environment to achieve HBCR safely and efficiently, which solved this unmet need for monitoring and supervision in tele-CR.

## 3. Information that Should be Monitored during Exercise Session in tele-CR

To provide appropriate exercise in tele-CR, two main types of information should be monitored: parameters that indicate the intensity of exercise performed by the participants and parameters that indicate the participant’s condition before, during, and after exercise.

For parameters that indicate the participant’s condition, the most commonly obtained parameter is heart rate. Heart rate is important for proper exercise control, and is a solely used parameter in the simplest tele-CR system [17]. Electrocardiographic information is also followed in many studies, which could show the situation about arrhythmia in addition to heart rate. Respiratory rate monitoring also helps check the burden of the exercise; it can be performed by checking the participant’s appearance without obtaining the information itself. Continuous blood pressure monitoring is difficult, and many studies have been limited to intermittent blood pressure measurement, such as before and after exercise [18]. Monitoring of oxygen saturation is helpful for participants who are susceptible to hypoxia. In some studies, body weight information has also been uploaded regularly [18]. A method of confirming the participant’s appearance during exercise through video chat is also useful, as it allows monitoring of the patient’s subjective symptoms and respiratory rate and to evaluate the burden on the participants based on their appearance. Conversely, as the amount of data increases, the complexity of the process and frequency of communication problems also increase. In addition, the available telemonitoring device is determined to some extent by exercise content.

On the other hand, the exercise intensity is sometimes difficult to monitor. The simplest way to measure exercise volume is to count the steps at a certain duration using a pedometer. Although accelerometers can more accurately assess activity levels, there are wide-ranged varieties in accelerometer accuracy [19, 20]. The participants’ geopositional data can be used to follow the scope of the activity [21]. To more accurately assess exercise intensity, a fixed exercise method needs to be developed, such as using an ergometer to adjust intensity.

## 4. Synchronous and Subsynchronous Telemonitoring in tele-CR

In tele-CR, one of the forms of data exchange is where the prepared patient data are given to a fixed web application and the healthcare professionals access the data as appropriate [22]. Telerehab III, developed in Europe and the United States, is an attempt to add a remote approach to outpatient HBCR [23]. Each patient wore an accelerometer and uploads the data at least every 2 weeks to a secure webpage. Based on the uploaded data, patients received weekly feedback *via* e-mail and/or short message service (SMS). In the WREN study, a randomized controlled trial of a web-based CR program with remote support, provided a web-based comprehensive CR program that enables participants to record and monitor their exercises and lifestyles [24]. Healthcare professionals check all queries from participants within 48 h of posting or contact them if they have not logged in for more than 7 days. These systems may cause a time lag of a few days to weeks between the time of data upload by the participants and the time of checking by experts [25]. For a system to improve participants’ safety, an immediate response is warranted. A real-time telemonitoring system was ultimately developed to solve these time lag problems and improve participant motivation and safety. By contrast, increasing the frequency of data upload by participants may have some demerits, which is the increase of the burden in the procedures, which makes it harder to continue tele-CR. Conversely, the decrease in participant data also reduces the burden on healthcare professionals, which may promote expansion of indicated cases.

**Fig. 1.**
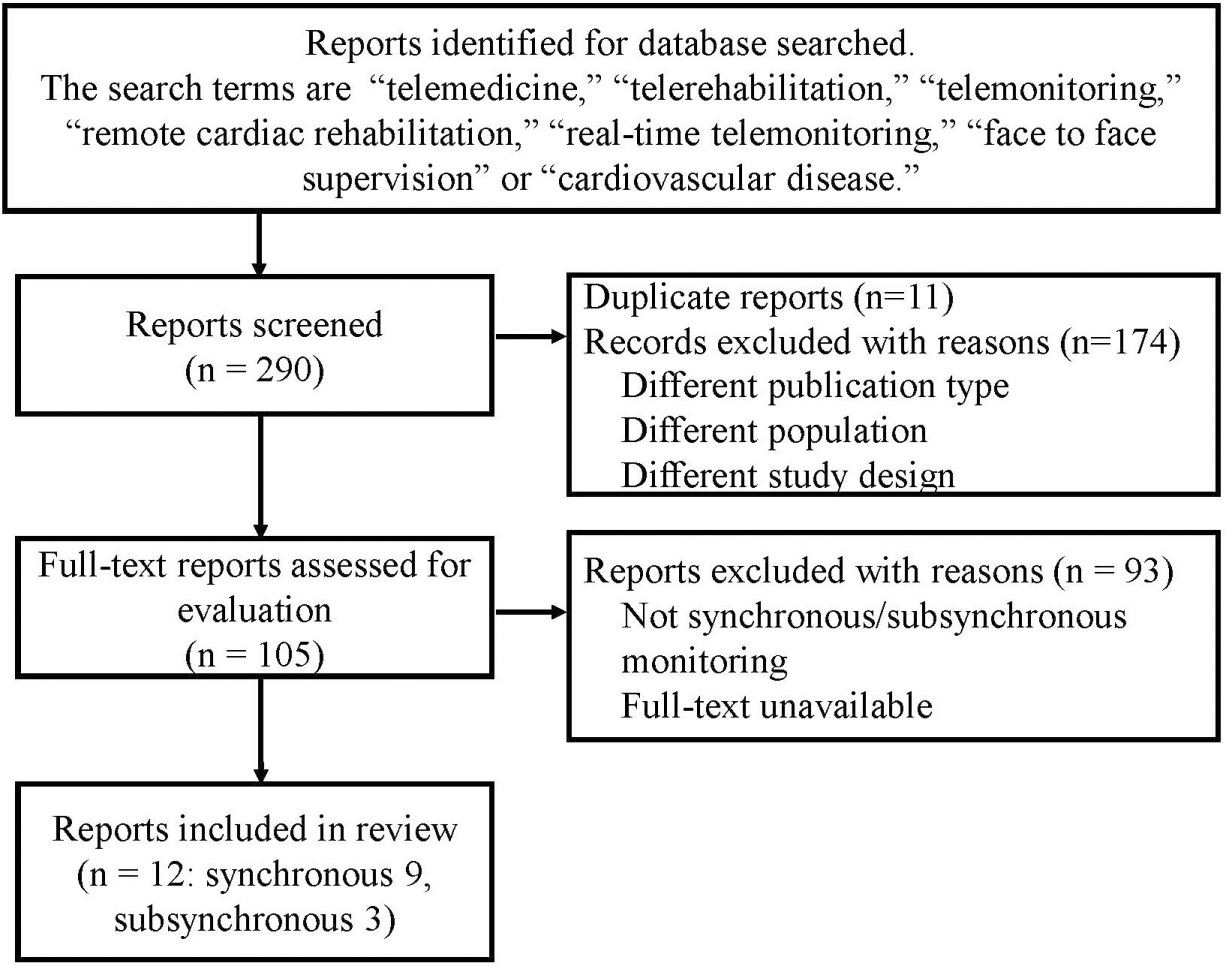
**Flowchart of the study selection process**.

We reviewed the literature to summarize the current situation in tele-CR with a focus on the methodology of patient telemonitoring. PubMed, Web of Science, and Scopus were searched to retrieve published studies that investigated tele-CR with monitoring of biological information, with the search terms “telemedicine”, “telerehabilitation”, “telemonitoring”, “remote cardiac rehabilitation”, “real-time telemonitoring”, “face to face supervision” or “cardiovascular disease”. After the removal of duplicates, articles were screened against the search terms. Only articles written in English were selected (Fig. 1). Almost all studies use their own system for telemonitoring in tele-CR (Table 1, Ref. [26, 27, 28, 29, 30, 31, 32, 33, 34, 35, 36, 37]). There are two methods for tele-CR system: “subsynchronous” and “synchronous (which corresponds to real-time)”. Subsynchronous telemonitoring refers to the transfer of data on vital signs, including heart rate, respiratory rate, and electrocardiogram, to the medical center at an appropriate time, such as before and after exercise training or when an unexpected event occurs, and the healthcare professionals can check patients’ parameters and the appropriateness of exercise training [26, 27, 28]. On the other hand, “synchronous” telemonitoring is the one with completely synchronous sharing of vital signs or electrocardiogram and even the speed and location of exercise training based on the Global Positioning System [29, 30]. Much previous research reported on vital telemonitoring *via* the Internet, smartphone, Bluetooth, or others. Skobel *et al*. [31] designed a closed-loop disease management system called the guided exercise (GEX) system, which had components to support participants exercise training. Participants wore a dedicated shirt with incorporated wireless sensors, electrocardiogram and heart rate during exercise, and the software provided immediate feedback via a smartphone [31]. In both synchronous and subsynchronous telemonitoring programs, the participants can contact the consultants before, during and after every exercise or if necessary *via* phone or e-mail [30, 32, 38].

**Table 1.** **Description of telerehabilitation studies including synchronous/subsynchronous telemonitoring and supervision**.

Reference	Age/Patients	Intervention (tele-CR system)	Exercise training	Telemonitoring	Supervision	Feedback	Outcomes
Taniguchi *et al*., 2021 [33]	age 76 ± 7	12-week tele-CR (original integrated tele-CR platform)	aerobic exercise using an IoT-equipped stationary ergometer	With a wireless ECG monitoring device	with videoconference	Real-time by therapists	Improvement of 6MWD
	HF						
Maddison *et al*., 2019 [29]	age 61 ± 12.7	12 weeks of individualized exercise prescription, and coaching plus education (platform comprised a smartphone and chest-worn wearable sensor)	walking or alternatives, three exercise sessions per week, duration and intensity level ranged from 30 to 60 minutes and 40%–65% heart rate reserve	HR, RR, single lead ECG and geopositional data via smartphone app	Real-time individualized audio coaching, feedback and social support	Real-time by therapists	V̇$O_{2}$ max was comparable in tele-CR and CBCR groups at 12 weeks
	CAD						
Tousignant *et al*., 2019 [35]	age 66	the 12-week cardiac program (telerehabilitation on the platform)	strengthening and flexibility exercises	sensors to monitor real-time vital signs (ECG, ${\mathrm{SpO}}_{2}$, HR)	bi-directional audio and video communication over the internet	Real-time by a nurse clinician	tendency to improve their physical capacities
	HF						
Rawstorn *et al*., 2018 [37]	age 61 ± 12.7	12 weeks of exercise training and coaching plus education (platform comprised a smartphone and chest-worn wearable sensor)	moderate to vigorous aerobic-based exercise for at least 30 minutes (preferably more), most days (≥5) of the week	HR, RR, single lead ECG and geopositional data via smartphone app	Real-time individualized audio coaching, feedback and social support	Real-time by therapists	usability and acceptability positively evaluated by most participants, 87% would choose tele-CR
	CAD						
Hwang *et al*., 2017 [34]	age 67	a 12-week, real-time exercise and education intervention (synchronous videoconferencing platform)	10-minute warm-up, 40-minutes of aerobic and strength exercises, and a 10-minute cool-down	BP, HR and oxygen saturation levels using the monitoring equipment	synchronous videoconferencing platform across the internet	Real-time by therapists	No significant difference of 6MWD between tele-CR and CBCR
	HF						
Peng *et al*., 2018 [36]	age 66.3	8-week home-based telehealth exercise training and education program using QQ and Wechat software (instant messaging service)	Aerobic resistance exercise training: walking and jogging via online webcam communication and supervision	Wearing HR monitor so that the rehabilitation doctors could adjust the training intensity	online webcam communication and supervision using QQ and Wechat software	Real-time by therapists	QOL and 6MWD were improved significantly in tele-CR group
	HF						
Skobel *et al*., 2017 [31]	age 58 ± 10	6-month follow up with smartphone-guided, a new training steering/feedback tool (Guided exercise system)	endurance training (e.g., cycling, walking), resistance training	a sensor for acquisition of vital signs and a smartphone for interaction with patients	The smartphone software provide immediate feedback with respect to training intensity	Real-time by software	peak ${\mathrm{VO}}_{2}$ improved more in the tele-CR group
	CAD						
Worringham *et al*., 2011 [30]	age 53.6 (42–67)	six weeks exercise program (original remote monitoring system)	average of three outdoor walking sessions per week	single-lead ECG, HR, GPS-based speed and location monitored with the belt with smartphone via Bluetooth	The patients would be contacted by phone in case of emergency by the doctor	Pre and post sessions by the exercise scientist via mobile phone, during exercise if needed.	Improvement of 6MWD, depression and QOL comparable to CBCR
	CAD						
Kouidi *et al*, 2006 [32]	age 56 ± 6.2	six-month exercise training programs in public gyms (telemedicine 12-lead ECG unit)	three exercise sessions at least per week, 90 minutes per session, including cycling, running, arm ergometry, predominately isotonic calisthenics and relaxation	Twelve-lead ECG was recorded and transmitted to the base by experienced trainers in gyms via telephone lines. ECG was evaluated by the medical staff at the base	Exercise training was performed under the instruction of exercise trainers in the gyms	cardiologists contacted the exercise trainer and provided the instructions for intervention	Successful ECG data was transmitted in 99.3%
	CVD						
Piotrowicz *et al*., 2020 [26]	age 62.6 ± 10.8/62.2 ± 10.2	tele-CR program: 1 week in hospital and 8 weeks at home (advanced monitoring systems)	endurance aerobic Nordic walking training, respiratory muscle training, and light resistance and strength exercises. 5 times weekly	tele-ECG, BP and body-weight via mobile telephone	Transmission of resting ECG to the monitoring center before, after the exercise training and in case of emergency	Before and after the exercise, and in case of emergency via mobile phone	tele-CR improved ${\mathrm{VO}}_{2}$ and QOL, but did not lead to the increase in percentage of days alive compared to UC
	HF						
Piotrowicz *et al*., 2015 [27]	age 54.4 ± 10.9/62.1 ± 12.5	a home-based telemonitored eight-week program with tele-CR set (EHO mini device and blood pressure measuring and weighing machine)	Nordic walking training. 5 times weekly	tele-ECG, BP and body-weight via mobile telephone	Transmission of resting ECG to the monitoring center before, after the exercise training and in case of emergency	Before and after the exercise, and in case of emergency via mobile phone	Nordic Walking resulted in significant improvement in peak ${\mathrm{VO}}_{2}$, 6MWD and QOL
	HF						
Piotrowicz *et al*., 2010 [28]	age 58.1 ± 10.2	home-based telemonitored CR (recording of ECG with the EHO 3 system)	Continuous walking training	ECG data from three pre-cordial leads monitored by the own system and transmittal via mobile phone to monitoring center	Transmission of resting ECG to the monitoring center before, after the exercise training and in case of emergency	Before and after the exercise, and in case of emergency via mobile phone	Significant improvement of peak ${\mathrm{VO}}_{2}$, 6MWD and QOL in tele-CR groups
	HF						

BP, blood pressure; CAD, coronary artery disease; CBCR, center-based cardiac rehabilitation; CVD, cardiovascular disease; ECG, electrocardiogram; GPS, Global Positioning System; HF, heart failure; HR, heart rate; IoT, Internet of Things; QOL, quality of life; ${\mathrm{SpO}}_{2}$, oxygen saturation; tele-CR, home-based cardiac rehabilitation with telemedicine; UC, usual care; ${\mathrm{VO}}_{2}$, oxygen consumption; 6MWD, 6-min walk distance.

The latest system of tele-CR includes real-time, face-to-face supervised, and telemonitoring exercise training [28, 29]. This system allows healthcare professionals to check the participants’ general conditions and provide precise exercise intensity, instructions and immediate feedback through videos. There are still a few previous reports, however, trials are increasing [33, 34].

## 5. Supervision during tele-CR 

**Fig. 2.**
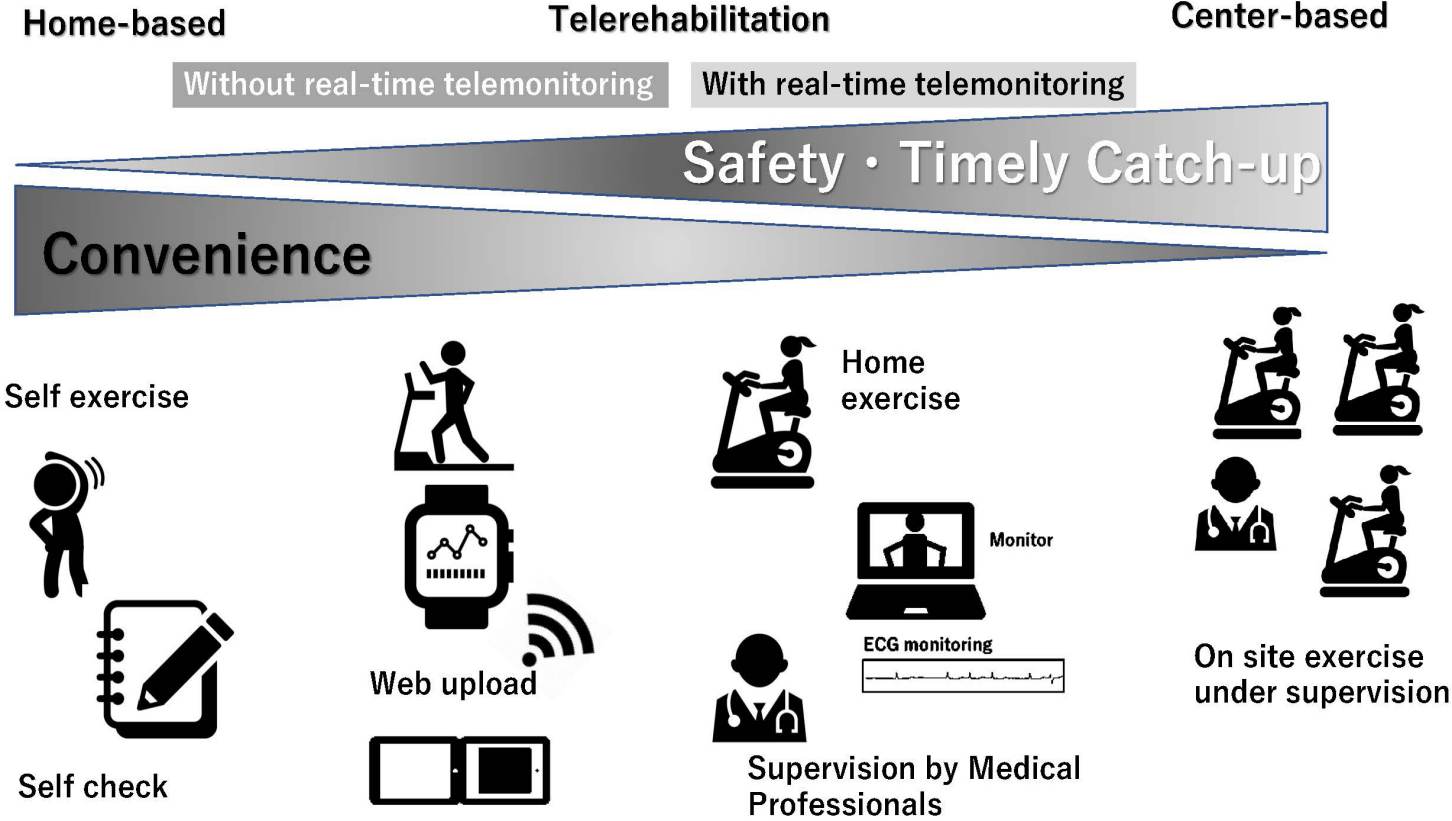
**Positioning of cardiac telerehabilitation from home-based to center-based cardiac rehabilitation**. ECG, electrocardiogram.

Supervision is most effective in the setting of real-time tele-CR. Telerehabilitation in heart failure patients (TELEREH-HF) [26], the largest study of tele-CR with a 9-week guideline-oriented tailor-made exercise program (1 week at the hospital and 8 weeks at home 5 times weekly; endurance aerobic Nordic walking training, respiratory muscle training, and light resistance and strength exercises) had their original tele-CR system for synchronous telemonitoring device for electrocardiogram and mobile phone for voice communication to healthcare professionals in exercise training. Healthcare professionals gave permission to start exercise after checking participants condition and electrocardiogram remotely. Recorded heart rate during exercise were also analyzed by monitoring healthcare professionals, and the safety, efficacy, and accuracy of a tailored patient’s rehabilitation program were assessed to adjust the exercise intensity appropriately [26]. In some studies, the participants can communicate with healthcare professionals *via* online videos or phone during exercise training [34, 35]. Kikuchi *et al*. [33] reported 12 weeks tele-CR using a stationary ergometer with synchronous real-time telemonitoring of heart rate and electrocardiogram via online videoconferencing system. Exercise intensity was set by the healthcare professionals for each session, targeting the optimal heart rate corresponding to the anaerobic threshold level determined based on the results of the baseline cardiovascular exercise test [33]. Real-time telemonitoring of heart rate and supervision make immediate adjustments of appropriate exercise intensity or cancellation decisions by healthcare professionals during worsening conditions [36]. Moreover, a group-based, video-linked tele-CR program has also emerged, which enables real-time interaction among all participants on the tele-CR system during exercise training [34]. This system provided more opportunities for patients to communicate with healthcare professionals and enjoy the group interaction or discussion on educational topics through the tele-CR system. This social connection may contribute to the high attendance rates for tele-CR. These aforementioned ideas will enable more effective and safer CR (Fig. 2).

By contrast, supervision in subsynchronous telemonitoring system is likely to be insufficient. It is necessary to use the subsynchronous system properly according to the characteristics of each case, such as a case where there is a margin for safety.

## 6. Safety and Effectiveness of tele-CR 

Safety is one of the most concerning points in tele-CR, and is evaluated in many studies [26, 30, 31, 32, 33, 34, 35, 36]. Heart rate, electrocardiogram and symptoms are useful parameters to assess participants’ condition and evaluate whether exercise intensity is appropriate or not during exercise training. Although some studies reported minor events, no major adverse events during exercise have been reported in tele-CR with either synchronous/subsynchronous telemonitoring [26, 30, 31, 32, 33, 34, 35, 36]. Maddison *et al*. [29] reported a high number of self-reported adverse events; however, most were mild or moderate and unrelated or possibly related to the interventions. The incidences of device troubles for real-time telemonitoring such as transmission failure, were also acceptable [30, 38, 37]. Moreover, a high satisfaction rate with tele-CR was reported [37]. One of the reasons was individualized exercise prescription and real-time telemonitoring and supervision by healthcare professionals, which were particularly valued as encouragement and motivation to adhere to exercise training. Although the definition of adherence is different between studies [26, 33, 34, 36, 37], attendance to prescribed exercise training or completion of follow-up are often used. Adherence to tele-CR is considered to be comparable to CBCR or usual care.

Many tele-CR studies set the primary outcome as exercise capacity measured using a 6-min walk test or oxygen consumption, physical strength, or quality of life. Almost all studies demonstrated improved exercise tolerance or capacity with tele-CR, comparable to the usual care or CBCR [29, 30, 31]. Hwang *et al*. [34] reported that 12-week tele-CR with real-time exercise using online videoconferencing software showed no significant difference in 6-minute walk distance compared to traditional CBCR, which were within the predetermined non-inferiority range. Improvement of quality of life (QOL) and 6-minute walk distance were reported to be sustained for 4 months in tele-CR group compared to control group [36]. These results suggest that tele-CR is a possible alternative for CBCR. On the other hand, little is known about the impact of tele-CR on prognosis. Piotrowicz E *et al*. [26] reported that the tele-CR program with subsynchronous telemonitoring did not decrease the mortality or readmission rates over a follow-up period of 14 to 26 months compared to usual care. Further research for the short and long-term effects of tele-CR on mortality or hospitalization is warranted. Whether improved exercise tolerance persists after tele-CR also needs to be investigated.

## 7. Cautions about tele-CR 

There are some concerns regarding the development of tele-CR in the real world. Eberly *et al*. [39] reported that the factors for less participation in telemedical care were females, non-English speakers and poorer patients during the COVID-19 era. Moreover, tele-CR was carried out by a limited number (8%) of facilities at the time of the Japanese nationwide survey conducted in 2020 [9]. Digital divide, which refers to the gap and unequal access to digital technology among older adults, is another emerging problem [40, 41]. In studies of tele-CR with real-time telemonitoring and supervision, the common age is approximately 50–60 years, and the oldest cohort was aged 76 [26, 27, 28, 29, 30, 31, 32, 33, 34, 35, 36, 37]. Regarding device handling, only those who could manipulate the device and system used in tele-CR were allowed to participate. The participants need to exhibit information technology literacy to be able to join tele-CR. Healthcare professionals need to check participants’ abilities in information technology literacy [42]. In addition, case-by-case consideration would be needed to determine whether to participate in tele-CR because it has little evidence for patients with severe heart failure or multiple comorbidities. In such patients, starting with CBCR may be recommended for close monitoring and prompt treatment in case of emergency. Recruitment of suitable patients is crucial, otherwise patients would feel uncomfortable with device equipment handling, arise safety concerns or be non-adherent. Although these problems are still unsolved, many trials about tele-CR, including ours, are now ongoing, and evidence is accumulating [43, 44]. Recently, research on artificial intelligence (AI) has increased [45]. In a not-so-distant future, tele-CR with AI might provide more stratified and tailor-made CR programs for participants with cardiovascular disease even for high-risk patients. tele-CR with telemonitoring is considered to be a strong platform that can be used as an alternative to the conventional outpatient CR system.

## 8. Conclusions

There is a lot of research of HBCR including tele-CR, however, the available research on real-time telemonitoring in tele-CR and supervision is still limited. Methodology of real-time telemonitoring in tele-CR was focused in this review. Each research had its own telemonitoring system and prompt response by healthcare professionals to achieve exercise training effectively and safely. Although there are barriers to the application of tele-CR, such as digital divide, tele-CR with synchronous/subsynchronous real-time telemonitoring and supervision is an effective and safe emerging model for HBCR in the current era.
